# Rivastigmine–Bambuterol Hybrids as Selective Butyrylcholinesterase Inhibitors

**DOI:** 10.3390/molecules29010072

**Published:** 2023-12-22

**Authors:** Jie Wu, Zekai Tan, Marco Pistolozzi, Wen Tan

**Affiliations:** 1 School of Food & Pharmaceutical Engineering, Zhaoqing University, Zhaoqing 526061, China; 2 Institute of Biomedical and Pharmaceutical Sciences, Guangdong University of Technology, Guangzhou 510006, China; 3120008972@mail2.gdut.edu.cn; 3 International School, Jinan University, Guangzhou 510632, China; pistolozzi@jnu.edu.cn; 4 Post-Doctoral Innovation Site, Jinan University Affiliation, Yuanzhi Health-Tech Inc., Hengqin District, Zhuhai 519000, China; 5 Kesi (Shandong) Innovation Service Inc., Heze Modern Medical Port, Mudan District, Heze 274009, China; 6 Jeffrey Cheah School of Medicine and Health Sciences, Monash University Malaysia, Bandar Sunway 47500, Malaysia

**Keywords:** Alzheimer’s disease, butyrylcholinesterase, rivastigmine, bambuterol, hybrid

## Abstract

Selective butyrylcholinesterase inhibitors are considered promising drug candidates for the treatment of Alzheimer’s disease. In this work, one rivastigmine–bambuterol hybrid (MTR-1) and fourteen of its analogues were synthesized, purified, and characterized. In vitro cholinesterase assays showed that all the compounds were more potent inhibitors of BChE when compared to AChE. Further investigations indicated that MTR-3 (IC_50(AChE)_ > 100,000 nM, IC_50(BChE)_ = 78 nM) was the best compound in the series, showing high butyrylcholinesterase selectivity and inhibition potency, the potential to permeate the blood–brain barrier, and longer-lasting BChE inhibition than bambuterol. These compounds could be used to discover novel specific BChE inhibitors for the treatment of Alzheimer’s disease.

## 1. Introduction

Alzheimer’s disease (AD) is a kind of neurodegenerative disease characterized by a loss of cholinergic neurons and decreased levels of the neurotransmitter acetylcholine (ACh) [1]. The reduction in ACh neurotransmission in specific regions of the brain is responsible for the cognitive impairment typical in AD patients. Hence, increasing ACh levels in the brain has been the most successful strategy for the treatment of AD so far [2]. Acetylcholinesterase (AChE) and butyrylcholinesterase (BChE), two enzymes classified as metabolic serine hydrolases, can terminate the action of ACh by catalyzing its hydrolysis. AChE inhibitors have been used to treat AD as this enzyme is considered the predominant cholinesterase (ChE) in the brain, whereas BChE is considered to have only a supportive role [3]. However, with the progression of AD, AChE levels progressively decrease while BChE levels increase, and BChE eventually becomes the most abundant ChE in the brain. Thus, it is likely that BChE activity assumes a more important role in cholinergic transmission in the late stages of the disease [4]. Molecules selectively targeting BChE are, therefore, potentially useful for the treatment of AD. In addition, selective BChE inhibitors might not present the common side effects of AChE inhibition, such as classic cholinergic toxicity [5].

**Figure 1:**
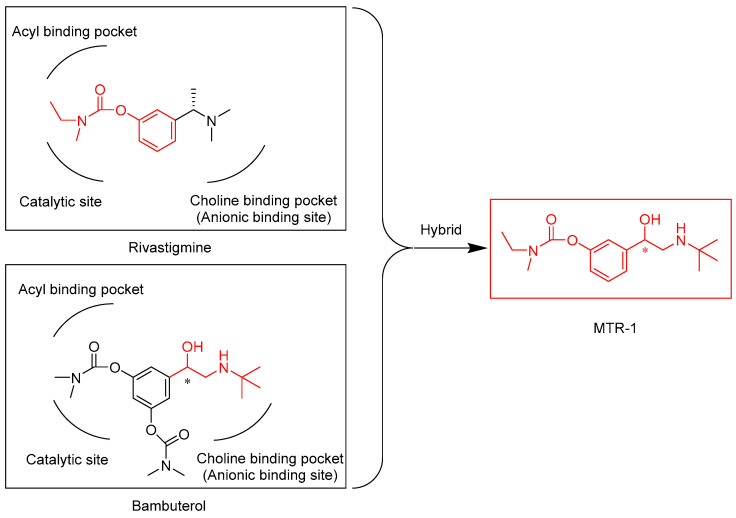
Structures of bambuterol, rivastigmine and MTR-1. * chiral center.

Rivastigmine, a dual inhibitor of AChE and BChE, has been used in patients affected by AD. Rivastigmine is classified as a pseudo-irreversible carbamate-type ChE inhibitor [6]. The interaction of AChE and BChE with rivastigmine results in the formation of (-)-S-3-[1-(dimethylamino)ethyl]phenol (NAP) and a carbamate-ChE adduct (inactive) as products. In a second step, the carbamate-ChE adduct is hydrolyzed, and ChE activity is restored [7]. Bambuterol, a bis-dimethylcarbamate prodrug of the β_2_-adrenoceptor agonist terbutaline, improves the symptoms of asthma and other affections characterized by bronchospasm. Bambuterol is metabolized by BChE to release its monocarbamate metabolite (MONO), which is further metabolized by BChE to form terbutaline. The mechanism in each step is the same described for rivastigmine. First, one carbamate group of bambuterol or MONO is transferred to the active site of BChE, resulting in the formation of MONO and a carbamate-BChE adduct or terbutaline and a carbamate-BChE adduct, respectively. Then, the enzyme activity is restored by water that removes the carbamyl group from the active site of BChE [8]. During this metabolism, bambuterol efficiently inhibits BChE with an IC_50_ of 3 × 10^−9^ M, whereas it has a much lower inhibitory effect on AChE (IC_50_ = 3 × 10^−5^ M) [9]. It has been reported that the high specificity of bambuterol for BChE compared to AChE depends on the difference in the structure of the choline binding site [10]. Inspired by the similarities of the structures of rivastigmine and bambuterol and their selectivity toward different ChEs, we wondered what the potency and selectivity of a rivastigmine–bambuterol hybrid (MTR-1, shown in Figure 1) would be. On this basis, MTR-1 and its analogues were prepared and evaluated as ChE inhibitors using AChE (electric eel AChE (elAChE)) and BChE (equine serum BChE (eqBChE)). Furthermore, an in silico molecular property analysis was performed to demonstrate the potential ability of the synthesized analogues to cross the blood–brain barrier. Finally, a complete kinetic study of eqBChE inhibition, including the measurement of the carbamylation and decarbamylation rate constants, was performed to demonstrate the inhibition mechanism of the best compound (MTR-3).

## 2. Results and Discussion

### 2.1. Chemistry

**Scheme 1:**
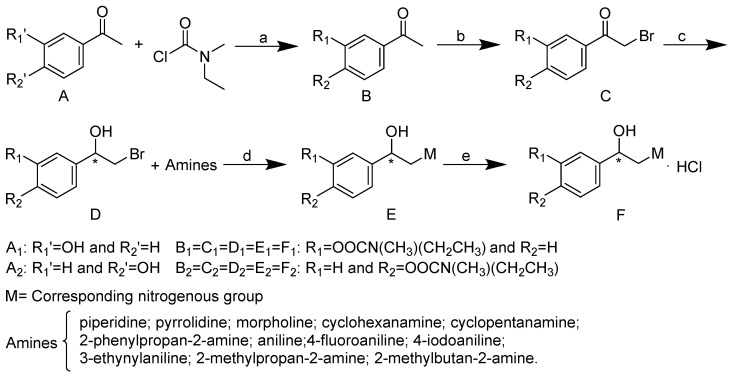
Synthesis of compounds. (a) Ethyl acetate, K_2_CO_3_•3/2H_2_O, K_2_CO_3_, pyridine, 70 °C; (b) ethyl acetate:CHCl_3_ = 1:1, CuBr_2_, 60 °C; (c) methanol, NaBH_4_, CH_2_Cl_2_; (d) isopropanol, 80 °C; (e) HCl in ethanol, 0 °C. * chiral center.

Bambuterol acts as a potent pseudo-substrate inhibitor of BChE. The difference in the structure of the choline binding sites between AChE and BChE contributes to the high specificity of bambuterol for BChE [10]. It is reasonable to predict that the ethanolamine side chain of bambuterol plays a key role in its high specificity for BChE. Rivastigmine is a dual inhibitor of AChE as well as BChE and has the same inhibitory mechanism and ChE binding sites as bambuterol [7]. On this basis, we hypothesized that the rivastigmine–bambuterol hybrid MTR-1 could have a much higher BChE selectivity than rivastigmine. Indeed, we found that MTR-1 has specific inhibitory activity towards BChE and could be regarded as a leading compound to find novel specific BChE inhibitors (Shown in Table 1). We then investigated modifications of MTR-1 involving the position of the carbamyl ester and substitutions of the tert-butylamine group, taking into account the structure–activity relationships of the bisdimethylcarbamate derivatives of metaproterenol and isoproterenol, as well as those of bambuterol analogues [11,12]. The synthesis of MTR-1 and its analogues is systematically represented in Scheme 1. We attempted the isolation of MTR-1 and MTR-2 as hydrochloride salts to enable purification via recrystallization according to the procedure used for the bambuterol analogues BD-6 and BD-11 reported in our previous work [12]. However, those two compounds did not form solid hydrochloride salts and were eventually purified by column chromatography. All the other analogues were directly purified by column chromatography without attempting to isolate them as solid salts.

### 2.2. Measurement of IC_50_

The inhibitory activities of the synthesized compounds on ChEs were evaluated utilizing elAChE and eqBChE. The IC_50_ values were determined by pre-incubating each enzyme with the compounds for 60 min before measuring the residual activity of the enzyme. The analysis of the IC_50_ values indicated that all the compounds were more potent inhibitors of eqBChE compared to elAChE (Table 1). We observed that the IC_50_ values for many of the compounds for elAChE were higher than 100 µM except for MTR-4, MTR-5, MTR-7 and MTR-15, which presented IC_50_ values lower than 100 µM, whereas the IC_50_ values of all the compounds for eqBChE were between 9 nM and 8700 nM. Most of the derivatives with the N-methyl-N-ethylcarbamyl group on the meta-position of the aromatic ring were more potent eqBChE inhibitors than MTR-1 except for MTR-6 and MTR-9. Changing the position of the N-methyl-N-ethylcarbamyl group on the aromatic ring from the meta-position to the para-position resulted in a decrease in inhibitory potency toward eqBChE. For tert-butyl amine derivatives, MTR-13 was a 22-fold less effective eqBChE inhibitor than MTR-1. For tert-amyl amine derivatives, MTR-14 was a ninefold less effective eqBChE inhibitor than MTR-2. For piperidine derivatives, MTR-15 was a fourfold less effective eqBChE inhibitor than MTR-3. Only for piperidine derivatives (MTR-3 and MTR-15) did changing the position of the N-methyl-N-ethylcarbamyl group on the aromatic ring from the meta-position to the para-position reduced the IC_50_ values toward elAChE below 100 μM. We reported that IC_50_ values of rivastigmine measured in the same conditions were 22.7 ± 4.8 µM and 0.785 ± 0.056 µM for elAChE and eqBChE, respectively [13]. Thus, most of the derivatives were more potent eqBChE inhibitors than rivastigmine, except for MTR-6, MTR-9, MTR-13 and MTR-14, but with much weaker inhibitory potency toward elAChE. Three derivatives (MTR-3, MTR-4 and MTR-5) were more effective at inhibiting eqBChE than bambuterol. Among those three derivatives, only MTR-3 showed an IC_50_ value for elAChE higher than 100 µM, similar to bambuterol.

### 2.3. In Silico Chemical Analysis of Molecular Properties

In drug development, the physicochemical properties of new drug candidates play an important role in determining whether they will move forward in the pipeline or not [14]. Drugs targeting the central nervous system must have the capacity to cross the blood–brain barrier (BBB) as minimum attribute, and in the early stages of development, this quality is often predicted using molecular descriptors, such as the partition coefficient (Log P) and the topological polar surface area (TPSA), as surrogate measures. In order to easily cross the BBB, compounds should have Log P = 2–5 and TPSA ≤ 70 Å^2^ [15,16]. Low polarity is also an important predictor of oral bioavailability [17]. In this work, an in silico molecular property analysis was performed using molinspiration software (www.molinspiration.com (accessed on 22 May 2020)). As shown in Table 2, most of the carbamates synthesized in this work had an estimated LogP = 2–5 except for MTR-6 and MTR-7, for which it was <2. All the compounds had TPSA values in the range between 53 Å^2^ and 62 Å^2^. Among the most potent eqBChE inhibitors (MTR-3, MTR-4 and MTR-5), MTR-3 was the one with the lowest TPSA value (53.01 Å^2^). All the other predicted values for MTR-3 were within the appropriate range based on Lipinski’s rule of five [18]. Overall, the in silico analysis indicated that MTR-3 is the most promising molecule of the series and deserves further study.

### 2.4. Kinetic Study of the Inhibition of eqBChE by MTR-3

**Figure 2:**
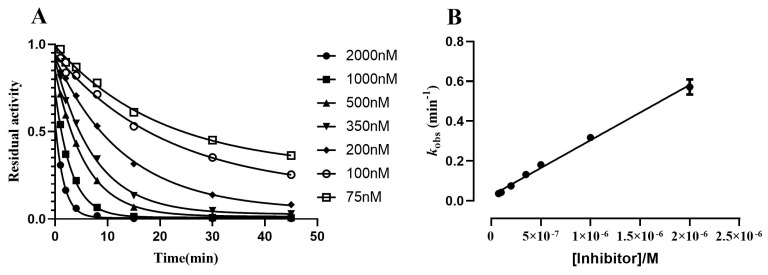
Time- and concentration-dependent inhibition of eqBChE (**A**) after pre-incubation with various concentrations of MTR-3. Linear correlation of the *k_obs_* values obtained for MTR-3 versus its respective concentrations ((**B**) eqBChE).

The inhibition constants (*k_I_*) for MTR-3 were calculated based on the equations reported earlier [8,12].

(1) $$R_{t}=v_{i}/v_{0}$$

(2) $$R_{t}=R_{0}\cdot \exp(-k_{obs}\cdot t)+R_{\infty }$$

(3) $$k_{obs}=k_{I}\cdot [I]$$

where *v_i_* is the eqBChE activity at set time points after pre-incubation with MTR-3, while *v*_0_ is the eqBChE activity at the same time points after pre-incubation with a blank solution, representing the maximum eqBChE activity. $R_{t}$, $R_{0}$ and $R_{\infty }$ are the residual fractional activities at time *t*, 0 and infinity, respectively. The pseudo-first order rate constant values (*k_obs_*) of MTR-3 were obtained by fitting the residual activity measured after different pre-incubation times to a one-phase exponential decay equation (Equation (2)). All data were analyzed as for a simple irreversible inhibition, and the second-order carbamylation rate constant (*k_I_*) was calculated using Equation (3). The inhibition of eqBChE by MTR-3 was time-dependent (i.e., the extent of the inhibition increased while increasing the pre-incubation time) and concentration-dependent (i.e., the extent of the inhibition increased while increasing the inhibitor concentration) (Figure 2A). The results obtained with eqBChE depicted a linear relationship between the concentration of MTR-3 and *k_obs_* (Figure 2B). The linearity of this relationship indicates that the range of concentration employed is below the affinity (KD) of the BChE-MTR-3 Michaelis complex; therefore, the fraction of the observed inhibition that can be attributed to the enzyme–inhibitor Michaelis complex is negligible. As shown in Table 3, we previously reported that the *k_I_*_1_ value of bambuterol measured under the same conditions was (1.44 ± 0.02) × 10^5^ M^−1^ min^−1^ for eqBChE. The *k_I_* value of MTR-3 for eqBChE ((2.78 ± 0.15) × 10^5^ M^−1^ min^−1^) was in the same order of magnitude of those of bambuterol, leading to the conclusion that MTR-3 and bambuterol have similar effectiveness at inhibiting BChE, consistent with the pattern of IC_50_ values.

### 2.5. Determination of the Decarbamylation Rate Constants of N-Methyl-N-ethyl-eqBChE, k_3_

**Figure 3:**
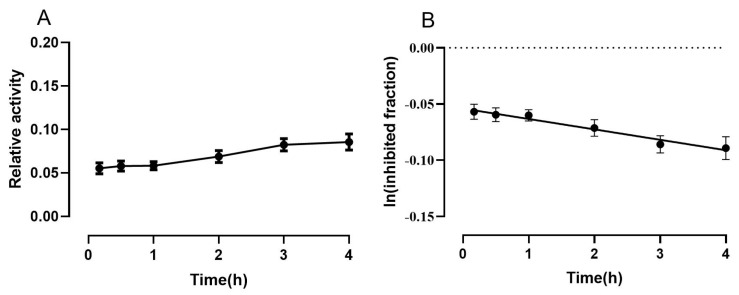
(**A**) Activity recovery of eqBChE after inhibition with MTR-3. (**B**) Decarbamylation kinetics of carbamylated eqBChE determined using Equation (4). Data are represented in the form of mean ± SD values (*n* = 3). The slope of the linear fitting corresponds to −*k*_3_.

The decarbamylation rate constant (*k*_3_) was calculated according to Equation (4) [8,12].

(4) $$\ln[1-(v_{i}/v_{0})]=-k_{3}\cdot t$$

where *v_i_* and *v*_0_ are the measured activities of eqBChE pre-incubated with MTR-3 or a blank solution at predetermined time points (*t*) after dilution. The slope of the linear data regression corresponds to −*k*_3_ (Figure 3B). The decarbamylation rate constants were obtained using a single concentration of MTR-3. This concentration was selected to be sufficiently high to provide a high initial fractional concentration of carbamylated BChE but sufficiently low to avoid the carbamylation of the enzyme by excess MTR-3 after dilution while monitoring the decarbamylation process. The inhibition of BChE by MTR-3 was found to be pseudo-irreversible in nature. It was previously observed that the *k*_3_ value of N, N-dimethyl-BChE is larger than that of N-methyl-N-ethyl-BChE for eqBChE [12]. Indeed, we found that the *k*_3_ value of MTR-3 (eqBChE: (0.0093 ± 0.0010) × h^−1^) was smaller than that of bambuterol (eqBChE: (0.0839 ± 0.0028) × h^−1^). As expected, the *k*_3_ value produced by MTR-3 was similar to that of the bambuterol analogue BD-11 (eqBChE: (0.0092 ± 0.0003) × h^−1^) in the same conditions, indicating that MTR-3 had the same long-term BChE inhibition as BD-11 due to the formation of an N-methyl-N-ethyl-BChE adduct during the catalytic process [12]. The decrease in *k*_3_ passing from methyl to ethyl as one of the alkyl groups on the carbamyl nitrogen is consistent with previous studies on inhibitors of human BChE, human AChE and elAChE, which showed a decrease in *k*_3_ as the group size increased [19,20,21].

## 3. Materials and Methods

### 3.1. Chemistry

^1^H-NMR spectra and ^13^C-NMR spectra were acquired with Bruker spectrometers. Chemical shifts were reported as parts per million (ppm) relative to TMS, used as an internal standard for both ^1^H NMR and ^13^C NMR. All NMR analyses were carried out at room temperature. Mass spectra were recorded on a Q-TOF mass spectrometer (Bruker, Bremen, Germany or Agilent Technologies Co., Ltd., Santa Clara, CA, USA) with an ESI source. The detection of TLC was performed using UV light at 254 nm and silica F254. The solvents used in all experiments were anhydrous unless otherwise specified.

### 3.2. General Procedures for the Preparation of Carbamates

#### 3.2.1. Method 1: Preparation of Compounds without Salt Formation (MTR-3 to MTR-15)

Compound A (44 mmol), K_2_CO_3_ (9 mmol), pyridine (0.5 mL) and K_2_CO_3_•3/2H_2_O (35 mmol) were dissolved in 60 mL of ethyl acetate, and the mixture was stirred at 70 °C. Ethyl(methyl)carbamic chloride (66 mmol) was dissolved in 40 mL of ethyl acetate, and the solution was added dropwise to this mixture at 70 °C. The process of the reaction was monitored via TLC. Water (60 mL) was then added to the reaction system and stirred at 70 °C for 2.5 h. After cooling the system down to room temperature, the organic phase was washed with a solution of 2% H_2_SO_4_ and water, dried over MgSO_4_ and filtered. The filtrate was evaporated to yield product B.

CuBr_2_ (2 equivalents) and product B (6 g) were dissolved in ethyl acetate (50 mL) and chloroform (50 mL). The reaction system was stirred at 60 °C and monitored via TLC. The solid residue was then removed, and the organic phase was washed five times using water. The organic phase was dried over MgSO_4_ and filtered again. Compound C was obtained by concentrating the filtrate.

NaBH_4_ (3 equivalents) and compound C (C_1_ = 3 g and C_2_ = 8 g) were dissolved in dichloromethane at room temperature. Methanol was added to the mixture at 0 °C, and the reaction system was stirred for 10 min. The reaction system was then stirred at 40 °C and monitored via TLC. NH_4_Cl solution (60 mL) was added to the mixture and stirred for 2 h at room temperature. The water phase was then extracted twice using dichloromethane and the combined dichloromethane was dried over MgSO_4_. Finally, the mixture was filtered and concentrated to yield product D.

Amines (2.5 to 3 equivalents) and D (0.5 g or 1 g) were dissolved in isopropanol and stirred at 80 °C. The reaction system was monitored via TLC. The resulting solution was then concentrated to yield a crude product. Dichloromethane (30 mL) was added to the product, and the mixture was washed with water. The organic phase was then dried over MgSO_4_ and filtered. The filtrate was finally concentrated to yield the product E. The crude product E was purified via flash column chromatography using ethyl acetate and petroleum ether or dichloromethane and methanol.

#### 3.2.2. Method 2: Preparation of MTR-1 and MTR-2 as Hydrochloride Salts

Hydrochloric acid in ethanol was added to a solution of crude product E in ethanol to adjust the solution to pH = 1. The reaction system was stirred at 0 °C for 4 h. The reaction solution was then concentrated and purified via flash column chromatography using dichloromethane and methanol as a mobile phase.

#### 3.2.3. 3-(2-(Tert-butylamino)-1-hydroxyethyl)phenyl ethyl(methyl)carbamate Hydrochloride (MTR-1)

Yield: 35%. ^1^H NMR (400 MHz, DMSO) δ = 7.39 (t, J = 7.8 Hz, 1H, H_arom_), 7.27 (d, J = 7.6 Hz, 1H, H_arom_), 7.18 (s, 1H, H_arom_), 7.06 (d, J = 8.2 Hz, 1H, H_arom_), 5.01 (d, J = 8.9 Hz, 1H, CH), 3.43 (dd, J = 13.9, 6.8 Hz, 1H, CON(CH_3_)(CH_2_CH_3_)), 3.31 (dd, J = 14.0, 6.9 Hz, 1H, CON(CH_3_)(CH_2_CH_3_)), 2.83–3.10 (m, 5H, CH_2_ and CON(CH_3_)(CH_2_CH_3_)), 1.31 (s, 9H, C(CH_3_)_3_), 1.15 (dt, J = 34.5, 7.0 Hz, 3H, CON(CH_3_)(CH_2_CH_3_)) ppm; ^13^C NMR (101 MHz, DMSO) δ = 154.09, 151.81, 143.95, 129.63, 123.24, 121.76, 119.85, 68.94, 56.62, 49.05, 43.95, 34.38, 34.05, 25.58, 13.56, 12.76 ppm; TOF MS ES(+)(*m*/*z*): calculated for C_16_H_27_N_2_O_3_ ([M+H]^+^) 295.2016, found 295.2011.

#### 3.2.4. 3-(1-Hydroxy-2-(tert-pentylamino)ethyl)phenyl Ethyl(methyl)carbamate hydrochloride (MTR-2)

Yield: 20%. ^1^H NMR (400 MHz, DMSO) δ = 9.36 (s, 1H, NH), 8.44 (s, 1H, HCl), 7.39 (t, J = 7.9 Hz, 1H, H_arom_), 7.27 (d, J = 7.7 Hz, 1H, H_arom_), 7.18 (s, 1H, H_arom_), 7.06 (dd, J = 8.0, 1.5 Hz, 1H, H_arom_), 6.27 (s, 1H, OH), 5.02 (d, J = 9.3 Hz, 1H, CH), 3.43 (dd, J = 14.0, 6.9 Hz, 1H, CON(CH_3_)(CH_2_CH_3_)), 3.28–3.34 (m, 1H, CON(CH_3_)(CH_2_CH_3_)), 2.98 (dd, J = 48.6, 14.4 Hz, 5H, CH_2_ and CON(CH_3_)(CH_2_CH_3_)), 1.68 (q, J = 7.5 Hz, 2H, CH_2_CH_3_), 1.26 (d, J = 1.5 Hz, 6H, C(CH_3_)_2_), 1.15 (dt, J = 14.0, 6.5 Hz, 3H, CON(CH_3_)(CH_2_CH_3_)), 0.88 (t, J = 7.5 Hz, 3H, CH_2_CH_3_) ppm; ^13^C NMR (101 MHz, DMSO) δ = 154.07, 151.79, 143.80, 129.62, 123.24, 121.76, 119.85, 68.81, 59.87, 48.61, 43.94, 34.38, 34.05, 30.28, 22.49, 22.39, 13.56, 12.75, 8.37 ppm; TOF MS ES(+)(*m/z*): calculated for C_17_H_29_N_2_O_3_ ([M +H]^+^) 309.2173, found 309.2171.

#### 3.2.5. 3-(1-Hydroxy-2-(piperidin-1-yl)ethyl)phenyl Ethyl(methyl)carbamate (MTR-3)

Yield: 47%. ^1^H NMR (400 MHz, CDCl_3_) δ = 7.31 (dd, J = 9.2, 6.5 Hz, 1H, H_arom_), 7.13–7.21 (m, 2H, H_arom_), 7.02 (d, J = 7.3 Hz, 1H, H_arom_), 4.73 (dd, J = 10.6, 3.4 Hz, 1H, CH), 3.39–3.50 (m, 2H, CON(CH_3_)(CH_2_CH_3_)), 2.95–3.09 (m, 3H, CON(CH_3_)(CH_2_CH_3_)), 2.69 (s, 2H, CH_2_), 2.34–2.55 (m, 4H, piperidine), 1.56–1.68 (m, 4H, piperidine), 1.47 (dd, J = 11.3, 5.6 Hz, 2H, piperidine), 1.18–1.26 (m, 3H, CON(CH_3_)(CH_2_CH_3_)) ppm; ^13^C NMR (101 MHz, CDCl_3_) δ = 151.66, 144.06, 129.04, 122.47, 120.65, 119.10, 68.27, 66.68, 54.43, 44.05, 26.43, 26.03, 24.19, 13.21, 12.46 ppm; TOF MS ES(+)(*m/z*): calculated for C_17_H_27_N_2_O_3_ ([M+H]^+^) 307.2016, found 307.2001.

#### 3.2.6. 3-(2-(Cyclohexylamino)-1-hydroxyethyl)phenyl Ethyl(methyl)carbamate (MTR-4)

Yield: 19%. ^1^H NMR (600 MHz, CDCl_3_) δ = 7.31 (t, J = 7.9 Hz, 1H, H_arom_), 7.18 (d, J = 7.7 Hz, 1H, H_arom_), 7.15 (s, 1H, H_arom_), 7.01 (d, J = 5.8 Hz, 1H, H_arom_), 4.76 (d, J = 8.6 Hz, 1H, CH), 3.46 (dd, J = 14.1, 7.0 Hz, 1H, CON(CH_3_)(CH_2_CH_3_)), 3.40 (dt, J = 14.1, 6.9 Hz, 1H, CON(CH_3_)(CH_2_CH_3_)), 3.09–2.94 (m, 4H, CON(CH_3_)(CH_2_CH_3_) and CH_2_), 2.72 (dd, J = 12.0, 9.5 Hz, 1H, CH_2_), 2.53 (t, J = 10.4 Hz, 1H, CH_ring_), 1.93 (d, J = 11.8 Hz, 2H, CH_2ring_), 1.77–1.71 (m, 2H, CH_2ring_), 1.60 (dd, J = 9.2, 3.4 Hz, 1H, CH_2ring_), 1.26–1.12 (m, 8H, CON(CH_3_)(CH_2_CH_3_) and CH_2ring_) ppm; ^13^C NMR (151 MHz, CDCl_3_) δ = 154.62, 154.44, 151.64, 144.16, 144.10, 129.16, 122.58, 122.53, 120.84, 119.17, 71.00, 56.83, 53.77, 44.08, 34.24, 33.81, 33.11, 32.83, 25.83, 24.93, 13.23, 12.48 ppm; TOF MS ES(+)(*m/z*): calculated for C_18_H_29_N_2_O_3_ ([M+H]^+^) 321.2173, found 321.2172.

#### 3.2.7. 3-(2-(Cyclopentylamino)-1-hydroxyethyl)phenyl Ethyl(methyl)carbamate (MTR-5)

Yield: 22%. ^1^H NMR (500 MHz, DMSO) δ = 7.31 (t, J = 7.8 Hz, 1H, H_arom_), 7.17 (d, J = 7.7 Hz, 1H, H_arom_), 7.07 (s, 1H, H_arom_), 6.96 (d, J = 7.9 Hz, 1H, H_arom_), 4.61 (dd, J = 8.6, 3.7 Hz, 1H, CH), 3.41 (dd, J = 13.5, 6.6 Hz, 1H, CON(CH_3_)(CH_2_CH_3_)), 3.30 (dd, J = 13.8, 6.9 Hz, 1H, CON(CH_3_)(CH_2_CH_3_)), 3.07–2.87 (m, 4H, CON(CH_3_)(CH_2_CH_3_) and CH_2_), 2.66 (dd, J = 11.9, 3.8 Hz, 1H, CH_2_), 2.57 (dd, J = 11.8, 8.8 Hz, 1H, CH_ring_), 1.77–1.66 (m, 2H, CH_2ring_), 1.65–1.55 (m, 2H, CH_2ring_), 1.51–1.41 (m, 2H, CH_2ring_), 1.29 (td, J = 12.9, 6.7 Hz, 2H, CH_2ring_), 1.14 (dt, J = 41.3, 6.9 Hz, 3H, CON(CH_3_)(CH_2_CH_3_)) ppm; ^13^C NMR (126 MHz, DMSO) δ = 154.18, 154.06, 151.61, 146.65, 129.16, 123.03, 120.72, 119.64, 71.51, 59.37, 56.59, 43.91, 34.35, 34.03, 32.94, 32.87, 24.04, 24.03, 13.54, 12.75 ppm; TOF MS ES(+)(*m/z*): calculated for C_17_H_27_N_2_O_3_ ([M+H]^+^) 307.2022, found 307.2021.

#### 3.2.8. 3-(1-Hydroxy-2-morpholinoethyl)phenyl Ethyl(methyl)carbamate (MTR-6)

Yield: 30%. ^1^H NMR (500 MHz, DMSO) δ = 7.31 (td, J = 7.8, 3.0 Hz, 1H, H_arom_), 7.18 (d, J = 7.7 Hz, 1H, H_arom_), 7.08 (s, 1H, H_arom_), 6.96 (dd, J = 8.0, 1.5 Hz, 1H, H_arom_), 5.12 (d, J = 3.9 Hz, 1H, OH), 4.72 (dt, J = 8.0, 4.0 Hz, 1H, CH), 3.58–3.54 (m, 4H, morpholine), 3.41 (dd, J = 13.5, 6.5 Hz, 1H, CON(CH_3_)(CH_2_CH_3_)), 3.32–3.27 (m, 1H, CON(CH_3_)(CH_2_CH_3_)), 2.95 (d, J = 62.0 Hz, 3H, CON(CH_3_)(CH_2_CH_3_)), 2.46 (dd, J = 12.6, 8.4 Hz, 5H, morpholine and CH_2_), 2.37 (dd, J = 12.7, 4.3 Hz, 1H, CH_2_), 1.14 (dd, J = 34.9, 7.0 Hz, 3H, CON(CH_3_)(CH_2_CH_3_)) ppm; ^13^C NMR (126 MHz, DMSO) δ = 154.18, 151.59, 146.76, 129.11, 123.18, 120.73, 119.80, 71.19, 69.65, 67.07, 66.87, 66.68, 62.57, 54.16, 51.34, 43.91, 34.36, 34.03, 13.55, 12.76 ppm; TOF MS ES(+)(*m/z*): calculated for C_16_H_25_N_2_O_4_ ([M+H]^+^) 309.1814, found 309.1811.

#### 3.2.9. 3-(1-Hydroxy-2-(pyrrolidin-1-yl)ethyl)phenyl Ethyl(methyl)carbamate (MTR-7)

Yield: 47%. ^1^H NMR (500 MHz, DMSO) δ = 7.36 (t, J = 7.8 Hz, 1H, H_arom_), 7.24 (d, J = 7.7 Hz, 1H, H_arom_), 7.14 (s, 1H, H_arom_), 7.03 (dd, J = 8.0, 1.4 Hz, 1H, H_arom_), 5.96 (s, 1H, OH), 4.92 (dd, J = 8.7, 3.8 Hz, 1H, CH), 3.42 (d, J = 7.0 Hz, 1H, CON(CH_3_)(CH_2_CH_3_)), 3.31 (d, J = 7.0 Hz, 1H, CON(CH_3_)(CH_2_CH_3_)), 3.11–2.89 (m, 9H, pyrrolidine, CON(CH_3_)(CH_2_CH_3_) and CH_2_), 1.86 (d, J = 6.1 Hz, 4H, pyrrolidine), 1.14 (dt, J = 42.6, 6.9 Hz, 3H, CON(CH_3_)(CH_2_CH_3_)) ppm. ^13^C NMR (126 MHz, DMSO) δ = 154.12, 153.99, 151.71, 144.80, 129.48, 123.16, 121.43, 119.85, 69.09, 62.01, 54.17, 43.93, 34.37, 34.04, 23.23, 13.55, 12.75 ppm; TOF MS ES(+)(*m/z*): calculated for C_16_H_25_N_2_O_3_ ([M+H]^+^) 293.1860, found 293.1859.

#### 3.2.10. 3-(1-Hydroxy-2-(phenylamino)ethyl)phenyl Ethyl(methyl)carbamate (MTR-8)

Yield: 15%. ^1^H NMR (600 MHz, DMSO) δ = 7.34 (t, J = 7.8 Hz, 1H, H_arom_), 7.24 (d, J = 7.7 Hz, 1H, H_arom_), 7.15 (s, 1H, H_arom_), 7.10 = 7.06 (m, 2H, H_arom_), 7.00 (d, J = 9.1 Hz, 1H, H_arom_), 6.64 (dd, J = 8.5, 0.8 Hz, 2H, H_arom_), 6.54 (t, J = 7.2 Hz, 1H, H_arom_), 5.57–5.49 (m, 2H, NH and OH), 4.75 (dt, J = 8.1, 4.2 Hz, 1H, CH), 3.46–3.41 (m, 1H, CON(CH_3_)(CH_2_CH_3_)), 3.32 (d, J = 7.0 Hz, 1H, CON(CH_3_)(CH_2_CH_3_)), 3.26–3.20 (m, 1H, CH_2_), 3.08 (dd, J = 17.0, 8.0 Hz, 1H, CH_2_), 2.97 (d, J = 74.9 Hz, 3H, CON(CH_3_)(CH_2_CH_3_)), 1.20–1.11 (m, 3H, CON(CH_3_)(CH_2_CH_3_)) ppm; ^13^C NMR (151 MHz, DMSO) δ = 154.19, 154.06, 151.68, 149.07, 146.31, 129.35, 129.23, 123.20, 120.91, 119.75, 116.24, 112.73, 70.75, 51.70, 43.94, 34.37, 34.04, 13.56, 12.77 ppm; TOF MS ES(+)(*m/z*): calculated for C_18_H_23_N_2_O_3_ ([M+H]^+^) 315.1703, found 315.1702.

#### 3.2.11. 3-(1-Hydroxy-2-((4-iodophenyl)amino)ethyl)phenyl Ethyl(methyl)carbamate (MTR-9)

Yield: 17%. ^1^H NMR (500 MHz, DMSO) δ = 7.33 (t, J = 7.9 Hz, 3H, H_arom_), 7.23 (s, 1H, H_arom_), 7.13 (s, 1H, H_arom_), 6.99 (d, J = 7.9 Hz, 1H, H_arom_), 6.50 (d, J = 8.7 Hz, 2H, H_arom_), 5.84 (s, 1H, NH), 5.56 (s, 1H, OH), 4.71 (s, 1H, CH), 3.42 (dd, J = 13.5, 6.5 Hz, 1H, CON(CH_3_)(CH_2_CH_3_)), 3.32–3.28 (m, 1H, CON(CH_3_)(CH_2_CH_3_)), 3.19 (d, J = 13.0 Hz, 1H, CH_2_), 3.07 (dd, J = 12.6, 8.4 Hz, 1H, CH_2_), 2.96 (d, J = 62.1 Hz, 3H, CON(CH_3_)(CH_2_CH_3_)), 1.18–1.09 (m, 3H, CON(CH_3_)(CH_2_CH_3_)) ppm; ^13^C NMR (126 MHz, DMSO) δ = 154.05, 151.67, 148.83, 146.12, 137.53, 129.23, 123.21, 120.95, 119.77, 115.38, 76.43, 70.66, 51.38, 43.94, 34.37, 34.04, 13.56, 12.77 ppm; TOF MS ES(+)(*m/z*): calculated for C_18_H_22_IN_2_O_3_ ([M+H]^+^) 441.0670, found 441.0672.

#### 3.2.12. 3-(2-((4-Fluorophenyl)amino)-1-hydroxyethyl)phenyl Ethyl(methyl)carbamate (MTR-10)

Yield: 25%. ^1^H NMR (600 MHz, DMSO) δ = 7.33 (t, J = 7.8 Hz, 1H, H_arom_), 7.23 (d, J = 7.7 Hz, 1H, H_arom_), 7.14 (s, 1H, H_arom_), 6.99 (d, J = 7.9 Hz, 1H, Ha_rom_), 6.93–6.87 (m, 2H, H_arom_), 6.65–6.60 (m, 2H, H_arom_), 5.54 (d, J = 4.5 Hz, 1H, NH), 5.48 (dd, J = 7.0, 4.8 Hz, 1H, OH), 4.77–4.69 (m, 1H, CH), 3.42 (dd, J = 13.7, 6.7 Hz, 1H, CON(CH_3_)(CH_2_CH_3_)), 3.31 (t, J = 6.9 Hz, 1H, CON(CH_3_)(CH_2_CH_3_)), 3.23–3.16 (m, 1H, CH_2_), 3.08–3.03 (m, 1H, CH_2_), 2.96 (d, J = 75.1 Hz, 3H, CON(CH_3_)(CH_2_CH_3_)), 1.20–1.09 (m, 3H, CON(CH_3_)(CH_2_CH_3_)) ppm; ^13^C NMR (126 MHz, DMSO) δ = 155.67, 153.83, 151.67, 146.26, 145.83, 129.21, 123.20, 120.91, 119.78, 115.74, 115.57, 113.49, 113.43, 70.77, 52.20, 43.93, 34.36, 34.04, 13.56, 12.76 ppm; TOF MS ES(+)(*m/z*): calculated for C_18_H_22_FN_2_O_3_ ([M+H]^+^) 333.1609, found 333.1609.

#### 3.2.13. 3-(1-Hydroxy-2-((2-phenylpropan-2-yl)amino)ethyl)phenyl Ethyl(methyl)carbamate (MTR-11)

Yield: 14%. ^1^H NMR (500 MHz, DMSO) δ = 7.40 (d, J = 7.3 Hz, 2H, H_arom_), 7.27 (td, J = 7.6, 4.0 Hz, 3H, H_arom_), 7.17 (t, J = 7.3 Hz, 1H, H_arom_), 7.08 (d, J = 7.7 Hz, 1H, H_arom_), 6.98 (s, 1H, H_arom_), 6.94 (d, J = 8.0 Hz, 1H, Ha_rom_), 4.58–4.51 (m, 1H, CH), 3.29 (dd, J = 14.0, 6.9 Hz, 2H, CON(CH_3_)(CH_2_CH_3_)), 2.94 (d, J = 59.6 Hz, 3H, CON(CH_3_)(CH_2_CH_3_)), 2.39–2.31 (m, 2H, CH_2_), 1.35 (d, J = 3.4 Hz, 6H, C(CH_3_)_2_), 1.19–1.07 (m, 3H, CON(CH_3_)(CH_2_CH_3_)) ppm; ^13^C NMR (126 MHz, DMSO) δ = 154.18, 151.53, 148.36, 146.57, 129.09, 128.43, 126.37, 126.20, 123.10, 120.70, 119.63, 72.51, 55.74, 51.53, 43.91, 34.34, 34.01, 29.95, 29.73, 13.52, 12.74 ppm; TOF MS ES(+)(*m/z*): calculated for C_21_H_29_N_2_O_3_ ([M+H]^+^) 357.2173, found 357.2174.

#### 3.2.14. 3-(2-((3-Ethynylphenyl)amino)-1-hydroxyethyl)phenyl Ethyl(methyl)carbamate (MTR-12)

Yield: 22%. ^1^H NMR (500 MHz, DMSO) δ = 7.33 (t, J = 7.8 Hz, 1H, H_arom_), 7.23 (d, J = 7.7 Hz, 1H, H_arom_), 7.14 (s, 1H, H_arom_), 7.06 (t, J = 7.8 Hz, 1H, H_arom_), 7.00 (d, J = 7.9 Hz, 1H, H_arom_), 6.72 (s, 1H, H_arom_), 6.68 (dd, J = 8.2, 1.7 Hz, 1H, H_arom_), 6.63 (d, J = 7.5 Hz, 1H, H_arom_), 5.79 (s, 1H, NH), 5.57 (s, 1H, OH), 4.72 (s, 1H, CH), 4.00 (s, 1H, C≡CH), 3.45–3.40 (m, 1H, CON(CH_3_)(CH_2_CH_3_)), 3.31 (d, J = 7.3 Hz, 1H, CON(CH_3_)(CH_2_CH_3_)), 3.22 (d, J = 13.0 Hz, 1H, CH_2_), 3.14–3.04 (m, 1H, CH_2_), 2.96 (d, J = 62.5 Hz, 3H, CON(CH_3_)(CH_2_CH_3_)), 1.19–1.10 (m, 3H, CON(CH_3_)(CH_2_CH_3_)) ppm; ^13^C NMR (126 MHz, DMSO) δ = 154.18, 151.67, 149.14, 146.13, 129.62, 129.23, 123.22, 122.52, 120.93, 119.79, 119.55, 115.40, 113.52, 84.95, 79.70, 70.75, 51.35, 43.92, 34.36, 34.04, 13.55, 12.76 ppm; TOF MS ES(+)(*m/z*): calculated for C_20_H_23_N_2_O_3_ ([M+H]^+^) 339.1703, found 339.1703.

#### 3.2.15. 4-(2-(Tert-butylamino)-1-hydroxyethyl)phenyl Ethyl(methyl)carbamate (MTR-13)

Yield: 25%. ^1^H NMR (400 MHz, DMSO) δ = 7.43 (d, J = 8.4 Hz, 2H, H_arom_), 7.12 (d, J = 8.3 Hz, 2H, H_arom_), 4.99 (d, J = 9.1 Hz, 1H, CH), 3.42 (dd, J = 13.9, 6.9 Hz, 1H, CON(CH_3_)(CH_2_CH_3_)), 3.26–3.34 (m, 1H, CON(CH_3_)(CH_2_CH_3_)), 2.85–3.08 (m, 5H, CH_2_ and CON(CH_3_)(CH_2_CH_3_)), 1.31 (s, 9H, C(CH_3_)_3_), 1.14 (dt, J = 34.0, 6.9 Hz, 3H, CON (CH_3_)(CH_2_CH_3_)) ppm; ^13^C NMR (101MHz, DMSO) δ = 154.14, 151.28, 139.13, 127.41, 122.24, 68.92, 56.61, 48.80, 43.94, 34.38, 34.05, 25.57, 13.54, 12.74 ppm; TOF MS ES(+)(*m/z*): calculated for C_16_H_27_N_2_O_3_ ([M+H]^+^) 295.2016; found 295.2003.

#### 3.2.16. 4-(1-Hydroxy-2-(tert-pentylamino)ethyl)phenyl Ethyl(methyl)carbamate (MTR-14)

Yield: 26%. ^1^H NMR (400 MHz, CDCl_3_) δ = 7.44 (d, J = 8.4 Hz, 2H, H_arom_), 7.09 (d, J = 7.0 Hz, 2H, H_arom_), 5.33 (d, J = 9.7 Hz, 1H, CH), 3.35–3.52 (m, 2H, CON(CH_3_)(CH_2_CH_3_)), 3.15 (d, J = 11.7 Hz, 1H, CH_2_), 3.02 (d, J = 30.8 Hz, 3H, CON(CH_3_)(CH_2_CH_3_)), 2.84–2.94 (m, 1H, CH_2_), 1.74 (q, J = 7.4 Hz, 2H, CH_2_CH_3_), 1.39 (d, J = 9.6 Hz, 6H, C(CH_3_)_2_), 1.18–1.26 (m, 3H, CON(CH_3_)(CH_2_CH_3_)), 1.00 (t, J = 7.5 Hz, 3H, CH_2_CH_3_) ppm; ^13^C NMR (101MHz, CDCl_3_) δ = 154.39, 151.20, 137.20, 126.69, 121.89, 68.57, 59.89, 50.14, 44.07, 34.23, 33.79, 31.21, 23.40, 23.37, 13.19, 12.44, 8.00 ppm; TOF MS ES(+)(*m/z*): calculated for C_17_H_29_N_2_O_3_ ([M+H]^+^) 309.2173; found 309.2157.

#### 3.2.17. 4-(1-Hydroxy-2-(piperidin-1-yl)ethyl)phenyl Ethyl(methyl)carbamate (MTR-15)

Yield: 45%. ^1^H NMR (400 MHz, CDCl_3_) δ = 7.35 (d, J = 8.3 Hz, 2H, H_arom_), 7.08 (d, J = 5.6 Hz, 2H, H_arom_), 4.74 (dd, J = 10.5, 3.1 Hz, 1H, CH), 3.38–3.51 (m, 2H, CON(CH_3_)(CH_2_CH_3_)), 3.02 (d, J = 30.8 Hz, 3H, CON(CH_3_)(CH_2_CH_3_)), 2.71 (s, 2H, CH_2_), 2.52–2.34 (m, 4H, piperidine), 1.59–1.67 (m, 4H, piperidine), 1.48 (d, J = 5.5 Hz, 2H, piperidine), 1.18–1.26 (m, 3H, CON(CH_3_)(CH_2_CH_3_)) ppm; ^13^C NMR (101MHz, CDCl_3_) δ = 150.79, 139.12, 129.73, 126.68, 121.58, 68.22, 66.85, 54.47, 44.06, 34.23, 33.81, 26.35, 25.99, 24.35, 24.16, 13.21, 12.47 ppm; TOF MS ES(+)(*m/z*): calculated for C_17_H_27_N_2_O_3_ ([M+H]^+^) 307.2016; found 307.2001.

### 3.3. Measurement of ChE Activity

The ChE inhibition abilities of the compounds were determined using a modified version of Ellman’s assay [8,12,13,22]. All the experiments were conducted in 96-well plates, and the readings were obtained using an EnSpire Multimode 2300 plate reader (Perkin Elmer, MA, USA) or a multimode microplate reader (Berthold Technologies). elAChE, eqBChE, S-acetylthiocholine iodide (ATCh), S-butyrylthiocholine iodide (BTCh) and 5-5′-dithiobis (2-nitrobenzoic) acid (DTNB) were purchased from Sigma-Aldrich (Shanghai, PRC). ATCh and BTCh stock solutions were freshly prepared in water. A stock solution of DTNB (Ellman’s reagent) was prepared fresh in a sodium phosphate buffer (100 mM, pH 7, 0.15% NaHCO_3_ *w/v*). Stock solutions of drugs were prepared in a solution of acetonitrile and water (*v*:*v* = 1:9). The pH for the measurements was 8.

#### 3.3.1. Ellman’s Assay with Modification

First, 25 μL aliquots of drug solutions at different concentrations were added to 200 μL of elAChE (0.04 U/mL) or eqBChE (0.1 U/mL) in a phosphate buffer (100 mM sodium phosphate buffer, pH 8), and the reaction system was pre-incubated at 37 °C for determined time periods. Then, 25 μL of a solution containing ATCh or BTCh (2.5 mM) and DTNB (3.4 mM) in the 100 mM sodium phosphate buffer was added to the reactions. Enzyme activity (*v_i_*) was determined by measuring the absorbance (412 nm) at 37 °C for 5 min. A sample incubated with 25 μL of a solution of acetonitrile and water (*v*:*v* = 1:9) without a drug was analyzed in parallel to confirm the maximum enzyme activity (*v*_0_). A sample containing phosphate buffer (pH 8) in place of the enzyme was analyzed for each drug concentration to account for the spontaneous conversion of the substrate into a product, and the values obtained were subtracted from each measured activity.

#### 3.3.2. Determination of IC_50_ Values

This method was based on the general measurement of ChE activity. Residual ChE activity was confirmed at 60 min of the pre-incubation of elAChE or eqBChE with seven different concentrations of drugs. The measurements were repeated three times for each concentration.

#### 3.3.3. Determination of *k_I_* Using eqBChE and MTR-3

The value of *k_I_* for MTR-3 with eqBChE was determined as previously reported [8,12,13]. EqBChE was pre-incubated with seven concentrations of MTR-3 and a vehicle for set time intervals (1 min, 2 min, 4 min, 8 min, 15 min, 30 min and 45 min), and the activities were detected as described in Section 3.3.1. The concentrations of MTR-3 were 2 × 10^−6^ M, 1 × 10^−6^ M, 5 × 10^−7^ M, 3.5 × 10^−7^ M, 2 × 10^−7^ M, 1 × 10^−7^ M and 7.5 × 10^−8^ M.

#### 3.3.4. Determination of *k*_3_ Using eqBChE and MTR-3

For measuring the *k*_3_ value of eqBChE, 2.5 μL of eqBChE (30 U/mL) and 2.5 μL of MTR-3 (1600 nM) were mixed and pre-incubated at 37 °C for 30 min. Then, a phosphate buffer (1.495 mL, pH 8, 37 °C) was added to each eqBChE-drug mixture (dilution 1:300). At set time points, each sample (225 μL) was added to a well of a 96-well plate containing 25 μL of a solution of BTCh (2.5 mM) and DTNB (3.4 mM) in a phosphate buffer (100 mM, pH 8). EqBChE activities (*v_i_*) were measured based on the method mentioned above (Section 3.3.1). For each set time point, a sample pre-incubated with a solution of acetonitrile and water (*v*:*v* = 1:9) without a drug was analyzed to confirm the maximum enzyme activity (*v*_0_). A phosphate buffer (100 mM, pH 8) was used as a blank to account for the spontaneous conversion of the substrate into a product, and the values obtained were subtracted from each measured activity.

### 3.4. Prediction of Molecular Properties

An in silico molecular property analysis was performed using molinspiration software (version 2018, www.molinspiration.com (accessed on 22 May 2020)), which is a free online service.

## 4. Conclusions

Rivastigmine and bambuterol are pseudo-irreversible carbamate-type ChE inhibitors. In this work, a rivastigmine–bambuterol hybrid (MTR-1) and fourteen MTR-1 analogues were synthesized, and their inhibitory activities against AChE and BChE were evaluated in vitro. The molecular properties of the fifteen compounds were predicted using molinspiration online servers. All the carbamates were found to be more potent inhibitors of BChE than AChE. Among these compounds, MTR-3 displayed excellent inhibition and selectivity towards BChE, as well as good potential to permeate the blood–brain barrier. We also found that MTR-3 acted as a longer-term BChE inhibitor with respect to bambuterol. These data showed that MTR-3 may be a useful compound for the discovery of novel AD therapeutical agents.

## Figures and Tables

**Table 1:** IC_50_ values of the prepared compounds for elAChE and eqBChE.

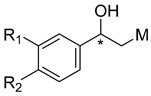					
Compound	R_1_	R_2_	M	elAChE IC_50_^a^ Mean ± SD (μM)	eqBChE IC_50_^a^ Mean ± SD (μM)
Rivastigmine				23 ± 5 ^b^	0.79 ± 0.06 ^b^
Bambuterol				>100 ^c^	0.119 ± 0.004 ^c^
MTR-1	OOCN(CH_3_)(CH_2_CH_3_)	H	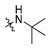	>100	0.37 ± 0.02
MTR-2	OOCN(CH_3_)(CH_2_CH_3_)	H	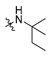	>100	0.17 ± 0.01
MTR-3	OOCN(CH_3_)(CH_2_CH_3_)	H	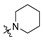	>100	0.078 ± 0.005
MTR-4	OOCN(CH_3_)(CH_2_CH_3_)	H	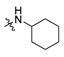	79 ± 4	0.0090 ± 0.0004
MTR-5	OOCN(CH_3_)(CH_2_CH_3_)	H	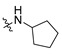	92 ± 7	0.074 ± 0.005
MTR-6	OOCN(CH_3_)(CH_2_CH_3_)	H	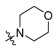	>100	2.5 ± 0.2
MTR-7	OOCN(CH_3_)(CH_2_CH_3_)	H	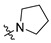	78.9 ± 0.9	0.21 ± 0.01
MTR-8	OOCN(CH_3_)(CH_2_CH_3_)	H	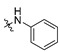	>100	0.32 ± 0.02
MTR-9	OOCN(CH_3_)(CH_2_CH_3_)	H	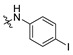	>100	1.73 ± 0.05
MTR-10	OOCN(CH_3_)(CH_2_CH_3_)	H	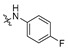	>100	0.192 ± 0.007
MTR-11	OOCN(CH_3_)(CH_2_CH_3_)	H	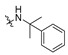	>100	0.323 ± 0.008
MTR-12	OOCN(CH_3_)(CH_2_CH_3_)	H	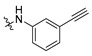	>100	0.156 ± 0.007
MTR-13	H	OOCN(CH_3_)(CH_2_CH_3_)	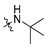	>100	8.7 ± 0.3
MTR-14	H	OOCN(CH_3_)(CH_2_CH_3_)	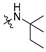	>100	1.7 ± 0.1
MTR-15	H	OOCN(CH_3_)(CH_2_CH_3_)	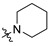	59 ± 16	0.42 ± 0.02

^a^ after pre-incubation with the enzyme for 60 min. ^b^ values obtained from [13]. ^c^ values obtained from [12]. * chiral center.

**Table 2:** In silico prediction of molecular properties of carbamates.

Code	MW	MiLogP	HBA	HBD	n_violations_	nROT	TPSA (Å^2^)	Volume
Rule	<500	≤5	≤10	≤5	≤1	-	≤70	-
Rivastigmine	250.34	2.28	4	0	0	5	32.78	254.01
MTR-1	294.39	2.07	5	2	0	7	61.80	294.96
MTR-2	308.42	2.58	5	2	0	8	61.80	311.76
MTR-3	306.41	2.04	5	1	0	6	53.01	302.32
MTR-4	320.43	2.79	5	2	0	7	61.80	318.76
MTR-5	306.41	2.29	5	2	0	7	61.80	301.96
MTR-6	308.38	0.98	6	1	0	6	62.24	294.50
MTR-7	292.38	1.53	5	1	0	6	53.01	285.52
MTR-8	314.38	2.58	5	2	0	7	61.80	300.18
MTR-9	440.28	3.67	5	2	0	7	61.80	324.17
MTR-10	332.38	2.75	5	2	0	7	61.80	305.11
MTR-11	356.47	3.29	5	2	0	8	61.80	349.80
MTR-12	338.41	2.32	5	2	0	7	61.80	322.44
MTR-13	294.39	2.10	5	2	0	7	61.80	294.96
MTR-14	308.42	2.60	5	2	0	8	61.80	311.76
MTR-15	306.41	2.06	5	1	0	6	53.01	302.32

MW: molecular weight; MiLogP: octanol–water partition coefficient; HBA: number of hydrogen bond acceptors; HBD: number of hydrogen bond donors; nviolations: violations of Lipinski’s rule; nROT: number of rotatable keys; TPSA: topological polar surface area.

**Table 3:** Summary of the kinetic parameters measured for the inhibition of eqBChE by MTR-3 and bambuterol.

Compound and Enzyme	*k_I_* (×10^5^ M^−1^ min^−1^) Mean ± SD	*k*_3_ (×h^−1^) Mean ± SD
MTR-3-eqBChE	2.78 ± 0.15	0.0093 ± 0.0010
Bambuterol-eqBChE	1.44 ± 0.02 ^a^	0.0839 ± 0.0028 ^a^

^a^ value obtained from [12].

## Data Availability

The data presented in this study are available wholly within the manuscript.
